# On Weak Exponential Expansiveness of Evolution Families in Banach Spaces

**DOI:** 10.1155/2013/284630

**Published:** 2013-07-25

**Authors:** Tian Yue, Xiao-qiu Song, Dong-qing Li

**Affiliations:** College of Science, China University of Mining and Technology, Xuzhou, Jiangsu 221008, China

## Abstract

The aim of this paper is to give several characterizations for the property of weak exponential expansiveness for evolution families in Banach spaces. Variants for weak exponential expansiveness of some well-known results in stability theory (Datko (1973), Rolewicz (1986), Ichikawa (1984), and Megan et al. (2003)) are obtained.

## 1. Introduction

In recent years, the exponential stability theory of one parameter semigroups of operators and evolution families has witnessed significant development. A number of long-standing open problems have been solved, and the theory seems to have obtained a certain degree of maturity. One of the most important results of the stability theory is due to Datko, who proved in 1970 in [1] that a strongly continuous semigroup of operators {*T*(*t*)}_*t*≥0_ is uniformly exponentially stable if and only if for each vector *x* from the Banach space *X* the function *t* → ||*T*(*t*)*x*|| lies in *L* ^2^(ℝ_+_). Later, Pazy generalizes the result in [2] for *L* ^*p*^(ℝ_+_), *p* ≥ 1. In 1973, Dakto [3] generalized the results above and proved that an evolutionary process *𝒰* = {*U*(*t*,*s*)}_*t*≥*s*≥0_ with uniform exponential growth is uniformly exponentially stable if and only if there exists an exponent *p* ≥ 1 such that sup⁡_*s*≥0_⁡∫_*s*_^*∞*^||*U*(*t*,*s*)*x*||^*p*^ *dt* < *∞*, for each *x* ∈ *X*. This result was improved by Rolewicz in 1986 (see [4]). In [5, 6], the authors generalized the results above in the case of *C*_0_-semigroups and evolutionary process, respectively, and presented a unified treatment in terms of Banach function spaces.

In the last few years, new concepts of exponential expansiveness and in particular, of exponential instability, have been introduced and characterized (see[7–14]). The cases of uniform exponential instability have been considered in [8] for evolution families and in [10] for linear skew-product flows.

In the present paper, we introduce the concept of weak exponential expansiveness for evolution families which is an extension of classical concept of exponential expansiveness. Our main objective is to give some characterizations for weak exponential expansiveness properties of evolution families in Banach spaces, and variants for weak exponential expansiveness of some well-known results in stability theory (Datko [3], Rolewicz [4], Ichikawa [15], and Megan et al. [8]) are obtained.

## 2. Preliminaries

Let *X* be a real or complex Banach space. The norm on *X* and on the space *B*(*X*) of all bounded linear operators on *X* will be denoted by ||·||.

**Definition 1**

A family *𝒰* = {*U*(*t*,*s*)}_*t*≥*s*≥0_ of bounded linear operators is called an evolution family if the following conditions are satisfied:

- *U*(*t*, *t*) = *I*, the identity operator on *X*, for all *t* ≥ 0;
- *U*(*t*, *r*)*U*(*r*, *s*) = *U*(*t*, *s*) for all *t* ≥ *r* ≥ *s* ≥ 0;
- there exist *M* ≥ 1 and *ω* > 0 such that ||*U*(*t*, *s*)*x*_0_|| ≤ *Me* ^*ω*(*t*−*s*)^||*x*_0_|| for all *t* ≥ *s* ≥ 0 and *x*_0_ ∈ *X*;
- for every *x*_0_ ∈ *X* and every *t*_0_ ≥ 0, the mapping *r* ↦ ||*U*(*r*, *t*_0_)*x*_0_|| is continuous on [*t*_0_, *∞*);
- for every *t* ≥ *s* ≥ 0, the operator *U*(*t*, *s*) is injective.

**Definition 2**

An evolution family *𝒰* = {*U*(*t*,*s*)}_*t*≥*s*≥0_ is called uniformly expansive if there exists a constant *N* > 0 such that

(1) $$\begin{array}{c} ||U\left(t,t_{0}\right)x_{0}||\geq N||U\left(r,t_{0}\right)x_{0}||, \end{array}$$

for all *t* ≥ *r* ≥ *t*_0_ ≥ 0 and *x*_0_ ∈ *X*.

**Definition 3**

An evolution family *𝒰* = {*U*(*t*,*s*)}_*t*≥*s*≥0_ is said to be uniformly exponentially expansive if there are *N*, *v* > 0 such that

(2) $$\begin{array}{c} ||U\left(t,t_{0}\right)x_{0}||\geq Ne^{v(t-r)}||U\left(r,t_{0}\right)x_{0}||, \end{array}$$

for all *t* ≥ *r* ≥ *t*_0_ ≥ 0 and *x*_0_ ∈ *X*.

**Remark 4**

It is obvious that an evolution family *𝒰* is uniformly exponentially expansive if and only if there are *N*, *v* > 0 such that

(3) $$\begin{array}{c} ||U\left(t,{\text{t}}_{0}\right)x_{0}||\geq Ne^{v(t-t_{0})}||x_{0}||, \end{array}$$

for all *t* ≥ *t*_0_ ≥ 0 and *x*_0_ ∈ *X*.

**Remark 5**

If the evolution family *𝒰* is uniformly exponentially expansive, then it is uniformly expansive. The converse is not necessarily valid. To show this, we consider the following example.

**Example 6**

Let *f* : ℝ_+_ → ℝ_+_ be a monotone increasing and bounded continuous function, *X* = ℝ_+_. The evolution family *𝒰* defined by

(4) $$\begin{array}{c} U\left(t,t_{0}\right)x_{0}=e^{f(t)-f(t_{0})}x_{0}, \end{array}$$

for all *t* ≥ *t*_0_ ≥ 0 and *x*_0_ ∈ *X*.

**Proof**

As a first step, we prove that *𝒰* is uniformly expansive. The evolution family *𝒰* satisfies the inequality

(5) ||U(t,t0)x0||=ef(t)−f(t0)|x0|≥ef(r)−f(t0)|x0|=N||U(r,t0)x0||,

for all *t* ≥ *r* ≥ *t*_0_ ≥ 0 and *x*_0_ ∈ *X*, where *N* = 1. Hence, *𝒰* is uniformly expansive.

As a second step, we prove that *𝒰* is not uniformly exponentially expansive. If we suppose that *𝒰* is uniformly exponentially expansive, then, by Remark 4, there exist some constants *N*, *v* > 0 such that

(6) $$\begin{array}{c} e^{f(t)-f(t_{0})}\geq Ne^{v(t-t_{0})}, \end{array}$$

for all *t* ≥ *t*_0_ ≥ 0.

In particular, for *t*_0_ = 0, we obtain *e* ^*f*(*t*)−*f*(0)^ ≥ *Ne* ^*vt*^, which is absurd for *t* → *∞*. Hence, *𝒰* is not uniformly exponentially expansive.

**Definition 7**

An evolution family *𝒰* = {*U*(*t*,*s*)}_*t*≥*s*≥0_ is called weakly exponentially expansive if there are *N*, *v* > 0 such that for all *x*_0_ ∈ *X* there exists *t*_0_ ≥ 0 with

(7) $$\begin{array}{c} ||U\left(t,t_{0}\right)x_{0}||\geq Ne^{v(t-r)}||U\left(r,t_{0}\right)x_{0}||, \end{array}$$

for all *t* ≥ *r* ≥ *t*_0_.

**Remark 8**

If the evolution family *𝒰* is uniformly exponentially expansive, then it is weakly exponentially expansive.

The following example shows that the converse is not valid.

**Example 9**

Let *X* = ℝ^2^ with the Euclidean norm. Consider the evolution family generated by the matrix *U*(*t*, *t*_0_) = *P*(*t*, *t*_0_)*Q*(*t*_0_), where

(8) $$\begin{array}{c} P\left(t,t_{0}\right)=\left(\begin{array}{cc} e^{t-t_{0}}\sin t & e^{-(t-t_{0})}\cos t\\ -e^{t-t_{0}}\cos t & e^{-(t-t_{0})}\sin t \end{array}\right),\\ Q\left(t_{0}\right)=\left(\begin{array}{cc} \cos t_{0} & \sin t_{0}\\ \sin t_{0} & -\cos t_{0} \end{array}\right). \end{array}$$

**Proof**

We divide the proof into two steps.

As a first step, we prove that *𝒰* is weakly exponentially expansive. For every *x*_0_ ∈ ℝ^2^, there exist *ρ* ≥ 0 and *t*_0_ ∈ [0,2*π*) such that *x*_0_ = (*ρ*cos⁡*t*_0_, *ρ*sin*t*_0_)^*T*^.

It is easy to see that

(9) U(t,t0)x0=P(t,t0)Q(t0)x0=P(t,t0)(ρ,0)T=(ρet−t0sint,−ρet−t0cos⁡t)T,

and hence

(10) $$\begin{array}{c} ||U\left(t,t_{0}\right)x_{0}||=\rho e^{t-t_{0}}\geq Ne^{t-r}||U\left(r,t_{0}\right)x_{0}||, \end{array}$$

for all *t* ≥ *r* ≥ *t*_0_ with *N* = 1, which shows that *𝒰* is weakly exponentially expansive.

As a second step, we prove that *𝒰* is not uniformly exponentially expansive. If we assume that *𝒰* is uniformly exponentially expansive, then there exist some constants *N*, *v* > 0 such that

(11) $$\begin{array}{c} ||U\left(t,t_{0}\right)y_{0}||\geq Ne^{v(t-r)}||U\left(r,t_{0}\right)y_{0}||, \end{array}$$

for all *t* ≥ *r* ≥ *t*_0_ ≥ 0 and *y*_0_ ∈ *X*.

In particular, for *y*_0_ = (sin*t*_0_, −cos⁡*t*_0_)^*T*^, we obtain

(12) U(t,t0)y0=P(t,t0)(0,1)T=(e−(t−t0)cos⁡t,e−(t−t0)sint)T,

and hence

(13) $$\begin{array}{c} ||U\left(t,t_{0}\right)y_{0}||=e^{-(t-t_{0})}=e^{-(t-r)}||U\left(r,t_{0}\right)y_{0}||, \end{array}$$

which shows that *𝒰* is not uniformly exponentially expansive.

## 3. The Main Results

**Theorem 10**

The following assertions are equivalent:

(i) *𝒰* is weakly exponentially expansive;
(ii) there are *δ* > 0 and *c* > 1 such that for every *x*_0_ ∈ *X* there exist *t*_0_ ≥ 0 and *h*_0_ ∈ (0, *δ*] with
  (14) $$\begin{array}{c} ||U\left(h_{0}+r,{\text{t}}_{0}\right)x_{0}||\geq c||U\left(r,t_{0}\right)x_{0}||, \end{array}$$
 for all *r* ≥ *t*_0_;
(iii) there exist *δ* > 0 and *c* > 1 such that for each *x*_0_ ∈ *X* there exists *t*_0_ ≥ 0 with the property that for every *r* ≥ *t*_0_ there is *h*_0_ ∈ (0, *δ*] with
  (15) $$\begin{array}{c} ||U\left(h_{0}+r,t_{0}\right)x_{0}||\geq c||U\left(r,t_{0}\right)x_{0}||. \end{array}$$

**Proof**

(i)*⇒*(ii) If *𝒰* is weakly exponentially expansive, then by Definition 7, there exist *N*, *v* > 0 such that for all *x*_0_ ∈ *X* there exists *t*_0_ ≥ 0 with the property

(16) $$\begin{array}{c} ||U\left(t,t_{0}\right)x_{0}||\geq Ne^{v(t-r)}||U\left(r,t_{0}\right)x_{0}||, \end{array}$$

for all *t* ≥ *r* ≥ *t*_0_. Let *δ* > 0 satisfy that *Ne* ^*vδ*^ > 1. Then, for *h*_0_ = *δ*, we have

(17) $$\begin{array}{c} ||U\left(r+h_{0},t_{0}\right)x_{0}||\geq Ne^{v\delta }||U\left(r,t_{0}\right)x_{0}||=c||U\left(r,t_{0}\right)x_{0}||, \end{array}$$

for all *r* ≥ *t*_0_.

(ii)*⇒*(iii) It is obvious.

(iii)*⇒*(i) We define *N* = 1/*Me* ^*ωδ*^ and *v* = ln⁡*c*/*δ*, where *δ* > 0 and *c* > 1 are given by (iii).

From (iii), it results that for each *x*_0_ ∈ *X*, there exists *t*_0_ ≥ 0 with the property that for every *r* ≥ *t*_0_ there is *h*_0_ ∈ (0, *δ*] such that

(18) $$\begin{array}{c} ||U\left(h_{0}+r,t_{0}\right)x_{0}||\geq c||U\left(r,t_{0}\right)x_{0}||. \end{array}$$

Let *r* ≥ *t*_0_, and we have that there is *h*_1_ ∈ (0, *δ*] with

(19) ||U(h1+h0+r,t0)x0||≥c||U(h0+r,t0)x0||≥c2||U(r,t0)x0||.

By induction, we have that

(20) $$\begin{array}{c} ||U\left(r_{n}+r,t_{0}\right)x_{0}||\geq c^{n}||U\left(r,t_{0}\right)x_{0}||,\;\forall n\in \mathbb{N}, \end{array}$$

where

(21) $$\begin{array}{c} r_{n}=\{\begin{array}{cc} 0, & n=0,\\ \overset{n-1}{\underset{i=0}{\sum }}h_{i}, & n\in {\mathbb{N}}^{*}, \end{array}\;h_{i}\in \left(0,\delta \right]. \end{array}$$

It is easy to see that (*r*_*n*_) is unbounded. In fact, if (*r*_*n*_) is bounded, then there exists r* ∈ ℝ with *r*_*n*_ → r* (*n* → *∞*). From the relation (20) and *c* > 1, it follows that

(22) $$\begin{array}{c} ||U\left(r+r^{*},t_{0}\right)x_{0}||\geq \underset{n\to \infty }{\lim}c^{n}||U\left(r,t_{0}\right)x_{0}||\to \infty , \end{array}$$

which is a contradiction because {*U*(*t*,*s*)}_*t*≥*s*≥0_⊆*B*(*X*).

So, (*r*_*n*_) is unbounded, and then for *t* ≥ *r*, there is *n* ∈ *ℕ* such that

(23) $$\begin{array}{c} r_{n}\leq t-r\leq r_{n+1}\leq \left(n+1\right)\delta . \end{array}$$

Then,

(24) ||U(rn+1+r,t0)x0||≤Meω(r+rn+1−t)||U(t,t0)x0||≤Meω(rn+1−rn)||U(t,t0)x0||≤Meωδ||U(t,t0)x0||,

and hence

(25) ||U(t,t0)x0||≥1Meωδ||U(r+rn+1,t0)x0||≥1Meωδcn+1||U(r,t0)x0||=1Meωδev(n+1)δ||U(r,t0)x0||≥Nev(t−r)||U(r,t0)x0||.

**Remark 11**

Theorem 10 can be considered a generalization of some results from uniform exponential instability proved in [8].

An important set in what follows is *ℱ*_1_, the set of all nondecreasing functions *F* : ℝ_+_ → ℝ_+_ with the properties:

(f1) *F*(*tr*) ≤ *F*(*t*)*F*(*r*), for all (*t*, *r*) ∈ ℝ_+_^2^_  _;
(f2) *F*(*t*) > 0, for every *t* > 0.

**Theorem 12**

An evolution family *𝒰* is weakly exponentially expansive if and only if there are *F* ∈ *ℱ*_1_ and *K* > 0 such that for every *x*_0_ ∈ *X*∖{0} there is *t*_0_ ≥ 0 with

(26) $$\begin{array}{c} \overset{\infty }{\underset{r}{\int }}F\left(\frac{1}{||U\left(\tau ,t_{0}\right)x_{0}||}\right)d\tau \leq KF\left(\frac{1}{||U\left(r,t_{0}\right)x_{0}||}\right), \end{array}$$

for all *r* ≥ *t*_0_.

**Proof**

*Necessity*. If *𝒰* is weakly exponentially expansive, then by Definition 7, there are *N*, *v* > 0 such that for all *x*_0_ ∈ *X*∖{0} there exists *t*_0_ ≥ 0 with

(27) ∫r∞1||U(τ,t0)x0||dτ≤∫r∞1Nev(τ−r)||U(r,t0)x0||dτ=1Nv||U(r,t0)x0||,

for all *r* ≥ *t*_0_.

Thus, the inequality (26) is satisfied for *F*(*t*) = *t* and *K* = 1/*Nv*.

*Sufficiency*. We assume for a contradiction that for all *δ* > 0 and *c* > 1 there exists *x*_0_ ∈ *X* such that for every *t*_0_ ≥ 0 there is *r* ≥ *t*_0_ with

(28) $$\begin{array}{c} ||U\left(t+r,t_{0}\right)x_{0}||<c||U\left(r,t_{0}\right)x_{0}||, \end{array}$$

for all *t* ∈ (0, *δ*].

In particular, for *δ* = *KF*(3) and *c* = 3, the inequality (28) implies

(29) F(3)∫r∞F(1||U(t,t0)x0||)dt  ≥∫r∞F(3||U(t,t0)x0||)dt  =∫0∞F(3||U(τ+r,t0)x0||)dτ  ≥∫0δF(3||U(τ+r,t0)x0||)dτ  >∫0δF(1||U(r,t0)x0||)dτ  =δF(1||U(r,t0)x0||),

which contradicts the inequality (26). This contradiction proves that *𝒰* is weakly exponentially expansive.

It makes sense to consider also the set *ℱ*_2_ all non-decreasing functions *F* : ℝ_+_ → ℝ_+_ with the properties:

(g1) *F*(*tr*) ≥ *F*(*t*)*F*(*r*), for all (*t*, *r*) ∈ ℝ_+_^2^_  _;
(g2) *F*(*t*) > 0, for every *t* > 0.

**Theorem 13**

An evolution family *𝒰* is weakly exponentially expansive if and only if there are *F* ∈ *ℱ*_2_ and *K* > 0 such that for every *x*_0_ ∈ *X*∖{0} there is *t*_0_ ≥ 0 with the relation (26).

**Proof**

*Necessity*. This is a simple verification for *F*(*t*) = *t*.

*Sufficiency*. It is similar to the proof of Theorem 12. Indeed, from (28) for *c* ∈ (1, +*∞*) and *δ* = 2*K*/*F*(1/*c*), we have

(30) ∫r∞F(1||U(t,t0)x0||)dt=∫0∞F(1||U(τ+r,t0)x0||)dτ≥∫0δF(1||U(τ+r,t0)x0||)dτ≥∫0δF(1c||U(r,t0)x0||)dτ≥δF(1c)F(1||U(r,t0)x0||)>KF(1||U(r,t0)x0||),

which contradicts the inequality (26).

**Remark 14**

The preceding theorems are variants for the case of weak exponential expansiveness property of a well-known theorem due to Rolewicz [4].

**Corollary 15**

An evolution family *𝒰* is weakly exponentially expansive if and only if there are *p* > 0 and *K* > 0 such that for all *x*_0_ ∈ *X*∖{0} there exists *t*_0_ ≥ 0 with

(31) $$\begin{array}{c} \overset{\infty }{\underset{r}{\int }}\frac{1}{{||U(t,t_{0})x_{0}||}^{p}}dt\leq K\frac{1}{{||U(r,t_{0})x_{0}||}^{p}}, \end{array}$$

for all *r* ≥ *t*_0_.

**Proof**

It is immediate from Theorem 12 for *F*(*t*) = *t* ^*p*^.

**Remark 16**

Corollary 15 is the version of a well-known theorem due to Datko [3], for the case of weak exponential expansiveness of evolution families.

In the following corollary, we give a discrete version of Theorems 12 and 13.

**Corollary 17**

An evolution family *𝒰* is weakly exponentially expansive if and only if there are *F* ∈ *ℱ*_1_ ∪ *ℱ*_2_ and *K* > 0 such that for all *x*_0_ ∈ *X*∖{0} there exists *t*_0_ ≥ 0 with

(32) $$\begin{array}{c} \overset{\infty }{\underset{n=0}{\sum }}F\left(\frac{1}{||U\left(r+n,t_{0}\right)x_{0}||}\right)\leq KF\left(\frac{1}{||U\left(r,t_{0}\right)x_{0}||}\right), \end{array}$$

for all *r* ≥ *t*_0_.

**Proof**

*Necessity*. This is a simple verification for *F*(*t*) = *t*.

*Sufficiency*. By Definition 1, we know that ||*U*(*r* + *τ*, *r* + *n*)|| ≤ *Me* ^*ω*^, *τ* ∈ [*n*, *n* + 1] and *τ* ↦ ||*U*(*r* + *τ*, *r* + *n*)|| is continuous on [*n*, *n* + 1] for all *n* ∈ *ℕ*, so there exist *L*_*n*_ > 0 such that

(33) $$\begin{array}{c} L_{n}=\underset{\tau \in [n,n+1]}{\min}||U\left(r+\tau ,r+n\right)||,\;n\in \mathbb{N}. \end{array}$$

Let *L* = inf⁡_*n*_⁡*L*_*n*_. We suppose that *F* ∈ *ℱ*_1_, and from (32), it results that

(34) ∫r∞F(1||U(t,t0)x0||)dt =∑n=0∞∫nn+1F(1||U(r+τ,r+n)U(r+n,t0)x0||)dτ ≤∑n=0∞∫nn+1F(1L||U(r+n,t0)x0||)dτ ≤F(1L)∑n=0∞F(1||U(r+n,t0)x0||) ≤KF(1L)F(1||U(r,t0)x0||),

for all *r* ≥ *t*_0_.

If *F* ∈ *ℱ*_2_, in a similar way we have

(35) F(L)∫r∞F(1||U(t,t0)x0||)dt ≤∫0∞F(L||U(r+τ,t0)x0||)dτ =∑n=0∞∫nn+1F(L||U(r+τ,r+n)U(r+n,t0)x0||)dτ ≤∑n=0∞∫nn+1F(1||U(r+n,t0)x0||)dτ ≤KF(1||U(r,t0)x0||),

for all *r* ≥ *t*_0_.

Applying Theorems 12 and 13, we conclude that *𝒰* is weakly exponentially expansive.

**Corollary 18**

An evolution family *𝒰* is weakly exponentially expansive if and only if there is *K* > 0 such that for all *x*_0_ ∈ *X*∖{0} there exists *t*_0_ ≥ 0 with

(36) $$\begin{array}{c} \overset{\infty }{\underset{n=0}{\sum }}\frac{1}{||U\left(r+n,t_{0}\right)x_{0}||}\leq K\frac{1}{||U\left(r,t_{0}\right)x_{0}||}, \end{array}$$

for all *r* ≥ *t*_0_.

Another characterization of the weak exponential expansiveness is given by the following.

**Theorem 19**

An evolution family *𝒰* is weakly exponentially expansive if and only if there are *K*, *α* > 0 such that for every *x*_0_ ∈ *X*∖{0} there is *t*_0_ ≥ 0 with

(37) $$\begin{array}{c} \frac{1}{t-r}\overset{t}{\underset{r}{\int }}e^{\alpha (\tau -r)}\frac{1}{||U\left(\tau ,t_{0}\right)x_{0}||}d\tau \leq K\frac{1}{||U\left(r,t_{0}\right)x_{0}||}, \end{array}$$

for all *t* > *r* ≥ *t*_0_.

**Proof**

*Necessity*. If *𝒰* is weakly exponentially expansive then by Definition 7, there are *N*, *v* > 0 such that for all *x*_0_ ∈ *X*∖{0} there is *t*_0_ > 0 with the property that for *α* = *v*/2 we have

(38) 1t−r∫rteα(τ−r)1||U(τ,t0)x0||dτ  ≤1N(t−r)∫rte−α(τ−r)dτ1||U(r,t0)x0||  =1−e−α(t−r)Nα(t−r)1||U(r,t0)x0||  ≤K1||U(r,t0)x0||,

for all *t* > *r* ≥ *t*_0_, where *K* = (1/*N*)sup⁡_*λ*>0_⁡((1 − *e* ^−*λ*^)/*λ*) < *∞*.

*Sufficiency*. Let *δ* > 0 be such that *e* ^*αδ*^ > 1 + 3*αδ* *K*, where *K* and *α* are given by (37). We suppose that *𝒰* is not weakly exponentially expansive. Then, by Theorem 10, for *c* = 3, there exists *x*_0_ ∈ *X* such that for all *t*_0_ ≥ 0 and all *u* ∈ (0, *δ*] there is *r* ≥ *t*_0_ with

(39) $$\begin{array}{c} ||U\left(u+r,t_{0}\right)x_{0}||<3||U\left(r,t_{0}\right)x_{0}||. \end{array}$$

Then, for *t* = *r* + *δ*, we have

(40) 1t−r∫rteα(τ−r)1||U(τ,t0)x0||dτ  =1δ∫0δeαu1||U(u+r,t0)x0||du  ≥13δ∫0δeαudu1||U(r,t0)x0||  =eαδ−13αδ1||U(r,t0)x0||  >K1||U(r,t0)x0||,

which contradicts the inequality (37), the proof is completed.
